# Practical Medicine and Surgery

**Published:** 1849-12

**Authors:** T. S. Bell

**Affiliations:** Louisville, Kentucky


Art. IV.—*Practical Medicine and Surgery.* By T. S. Bell, M. D., of Louisville, Ky.

In the November number of this Journal, I gave a programme of the state of anesthesia, with a view of calling the attention of medical men to the necessity of prudence in the use of such powerful agents as chloroform and ether. It is a source of no ordinary satisfaction to know that those remarks have been received in the spirit in

---

which they were made, and it is hoped that medical observers will be induced to report faithfully their experience with chloroform and ether. The following extract of a letter from a skilful and judicious practitioner of medicine, is a model of its kind, and we are sure the readers of this Journal will peruse its statements on the use of chloroform with much gratification. The object of the remarks on "Chloroform in Parturition," already referred to, was to point out the abuses of a perilous medicine, and thus lead to the proper use of it. The letter below will, to a considerable extent, contribute to directing the mind of practitioners to the proper use of chloroform :

"You have come to the rescue, and seconded the efforts to calm the undue excitement, and to restore reason to her just supremacy, in a manner that cannot fail to do good, and I doubt not will have the effect to arrest attention, and recall many to the less hazardous paths of prudence.

"Yet there are cases of parturition in which anesthesia is, beyond all question, of great utility, and the desideratum is some certain and definite rule, under which the practitioner will be justified in administering the agents calculated to induce it. I have sometimes been urgently solicited to administer letheon and chloroform by females alive to all the intense sufferings which they but too well knew awaited them, and who, under the dreadful apprehensions that disturbed their excited imaginations, were willing to incur almost any hazard that promised the least alleviation. To these I endeavored to represent fairly the dangers incident to the practice—the accidents that have unavoidably occurred, and the utter impossibility of ascertaining the conditions that secure immunity. Some have been deterred from their purpose ; but others, less timid, have insisted, and I have been induced to give the chloroform. I complied in these cases, not without much apprehension, until mature reflection, and close and anxious observation led me to the adoption of a single rule for my future guidance.

With much pleasure, I recognize that rule, and what I regard as the correct principle, in the following observation of your own: "for the mere oblivion of feeling, the peril of anesthetics is too great, and those who administer them should always find some apology in the peril of the patient without them." Relying on this as a wholesome truth, I never resort to anesthetics simply for the purpose of obviating pain. I employ them only when I deem them required by some peculiarity of the case, and to obviate some misfortune more threatening to health or life than the remedy. Under this rule, anesthesia may be resorted to with propriety in most difficult cases, requiring manual interference, or the use of instruments, and it is also broad enough to include all those whose experience has demonstrated that, owing to idiosyncrasy or from other causes, the shock inflicted on the nervous system, by the throes of labor, is such as seriously to impair the constitution, and eventually to endanger life. In such cases I believe we are fully authorized to appeal to the most heroic remedies that science has placed at our disposal, and he is recreant to duty who shrinks from the responsibility involved. To illustrate the latter position, and for the purpose of exhibiting the immense benefit that anesthetics are capable of conferring, when properly applied, I will relate briefly, the history of a single case that has occurred in my practice.

"Mrs. ——, about 25 years old, is the mother of six children. My professional intercourse with her commenced in March, 1847, shortly after her fourth confinement. I found her extremely feeble, and suffering intensely from neuralgia, generally affecting the head, but sometimes the back or limbs. I learned that she had been uniformly attended by an experienced and judicious accoucheur, but that, in spite of all the care which his own solicitude and the intensity of her agony elicited, she was, at the termination of each period, almost exhausted —appetite null, digestion impaired, and for months she continued to languish through sleepless nights and days of excruciating pain. After the lapse of three or four months, she would slowly rally, until about the period of a subsequent confinement, she was restored to tolerable health, to be again broken by the pains of childbirth.—Each period of confinement found her more debilitated than at the previous one, and less able to bear the increasing pangs that were rapidly and surely wasting the powers of life, and hastening the victim to a premature grave.

"At this time the child was necessarily abandoned to the care of a wet-nurse, while the mother was put upon a tonic course of treatment—temporary ease being obtained only by large and repeated opiates. It was only after three months, that the sufferer was restored to a degree of health compatible with a resumption of her domestic duties, which, however, continued to be performed in all the pain and discomfort known and appreciated only by those who endure them.

"In June, 1848, the same lady was again brought to bed, with her fifth child, and I was summoned to attend her. Earnestly pressed, by the lady and her husband, I reluctantly consented to the use of chloroform. After a labor of a few hours duration, she was safely delivered of a fine child, and very soon, after being made comfortable in bed, fell into a sound and refreshing slumber, from which she awakened expressing herself as having never felt better, and with mingled surprise and joy beaming in her countenance, exclaimed that she was entirely free from all those terrible sensations, that had ever before, on like occasions, proved so excruciating as to be far more dreaded by her than even the worst pains of labor. Subsequent to former labors, the most liberal use of opiates, in every form, had been resorted to, with little alleviation of her extreme anguish, and seldom with the effect to procure sleep. No untoward event happened on this occasion, and the lady was quite restored at the end of two weeks, and she continues to enjoy better health than at any time since her first lying in.

In September, 1849, I was again requested to attend this lady, and at the same time, received the assurance that no consideration would induce her to dispense with chloroform. Nor did I attempt to dissuade her from her purpose, being fully convinced that the advantage she had obtained from it justified her in repeating the experiment and amply compensated for any hazard that its use involved. She was speedily delivered, with little pain at any time, and none at the fearful moment of the exit of the head. I am happy to say that her recovery was as rapid and complete as in the most favored cases.

"Her health continued to improve, and she is at this time a healthy, happy mother, free from all the pangs that for years embittered life, and rendered her incapable of enjoying the society of her family and friends. To chloroform she attributes all the credit of her restoration to health, and I cannot gainsay her judgement.

"Averse as I am to the use of anesthetics in ordinary labors, I think they become exceedingly valuable in cases like that which I have thus briefly related, and that the accoucheur is in duty bound to avail himself of the advantages they offer.

"The first child lived in good health to the age of five months, and died, after a few days illness, of an acute disease. The last is now living, and is a remarkably sprightly, healthy, promising child.

Yours, W. S. CHIPLEY.

*Fracture of the Cranium—Concussion and Compression of the Brain.*—On the subject of injuries of the head Hennen says: "The young surgeon who, for the first time, witnesses a series of injuries of this description, will at every step have something to unlearn; he will find symptoms so complicated, contradictory, and insufficient to give any rational clue to their causes; diagnostics, of the truth of which he had read himself into a conviction, so totally unsupported by the results of practice; and the sympathies he was led to look for as infallible accompaniments of certain states of disease, so often wanting altogether, that he will possibly be inclined to relinquish the hope of ever arriving at a correct theory, or, at least, he will enter the clinical ward with the pride of science considerably reduced." No one can read Mr. Hennen's admirable reports of his cases, without realizing the accuracy of these remarks.

There is, in this state of things, an urgent demand for the numerical method in the detail of injuries of the head. When Mr. Thompson asserts that irregularity of the pulse is a frequent attendant upon compression of the brain, and Mr. Abernethy declares that intermission of the pulse is less frequent in compression than in concussion of the brain, we naturally feel a desire to learn the circumstances under which these adverse observations were made. When that mode of detailing facts, adopted by Louis, is applied carefully to injuries of the head, a great deal of the ambiguity and perplexity that now attends upon the diagnosis of these injuries will be removed. ed. Our experience, which is quite limited in these cases coincides with the observation of Mr. Thompson, but we differ from Mr. Abernethy with great reluctance.

As a contribution to this department of surgery we offer the following case. It presents some points of interest.

James Smith, aged about thirty-five years, a stout, hearty man, somewhat given to drink, was, on the twenty third of last October, by the violent plunging of his horse, thrown over the head of the animal, and the back of his head struck the rough pavement of Fulton street. He was stunned very much by the fall, but soon recovered, and when taken home walked, with a little assistance, from the side pavement up a steep bank, and from there, up stairs to his bed room. As he passed the negro woman belonging to the family he spake to her in a lively, good humored way, and asked her if she knew him. A neighboring physician was called to the case, and upon ascertaining the violence used, and the external injury inflicted, he was surprised at the absence of grave symptoms. This gentleman summoned his partner to the case soon after he saw it first, and the symptoms denoted nothing more than concussion, and a very judicious prescription was ordered. About an hour after this, the case was placed under my charge, and after a long and minute examination, I was unable to discover anything more than concussion of the brain. The skin was pale and cool, the pulse regular but oppressed, the breathing sometimes hurried, at others tranquil. The patient talked without difficulty, at times delirious, at others perfectly rational. His greatest trouble was to tell how he had received the injury. He could occasionally remember that a horse was connected with it, at other times he was confident that he had been struck with a bar of iron.

The external injuries were a triangular wound of the integuments over the central portion of the suture that unites the occipital and parietal bones. In front there was a deep gash in the inferior portion of the chin. The region of the scalp around the wound of the occipital integuments, was, under my direction, shaved, but after a careful examination I was unable to detect a depression, and entertained some hope that there was no fracture.—The pericranium was not cut, and I did not feel satisfied, by the symptoms, that there was any necessity for dividing that membrane: The judicious rule of Mr. Pott governed the decision. I had strong suspicions that more mischief had been done than was apparent, but I determined to watch the reaction and the general character of the case very closely. The suspicion of mischief was founded on the persistence of the effects of the concussion. There was little cause for suspecting compression; there was no approach to general insensibility ; the eyes acted naturally ; the pupils were obedient to their nature, contracting when exposed to the light, and dilating when it was withdrawn. The retina was sensible ; the limbs were not relaxed, nor the breathing stertorous. The presence of these symptoms is considered indicative of compression.

In order to assist the reluctant reaction, I directed five grains of the carbonate of ammonia to be given every two hours, and brandy panada whenever he could be induced to take it. The last prescription I considercd essential on account of the habits of the patient. My first visit to the case was between two and three o'clock, P. M. on the 23d. At six o'clock I saw him again, and found no material alteration, except that reaction was going on. At ten o'clock I was summoned to the case on account of several spasms, and the case was thus made additionally complicated. If there was any pressure, why had it been completely dormant? If the spasms denoted pressure, there was also reason to fear laceration of the substance of the brain. But the other symptoms had not changed. Just before the visit, my patient rose from his bed and walked to the fireplacc. After sitting some time he rose and returned to bed without aid. He answered my questions without any difficulty, and with sanity. I found that he had been unable to retain the brandy panada on his stomach, and I determined to purge him actively, for the relief of the spasms. After I made the prescription, he bade me good-night in a natural tone of voice.

Early on the morning of the 24th, I called on my patient, and found the most ample evidence of compression. What this evidence was, may be understood by the symp toms of compression detailed above. A peculiar motion of the hands and shoulders satisffed me so perfectly of the locality of the pressure, that I at once predicted that it was at the base of the brain, extending to the upper portion of the spinal column. I called Dr. J. B. Flint to the case, and a resort to the trephine was determined upon. On cutting the pericranium, a clean, undepressed fracture was found in the occipital bone, and, upon trepanning it, a mass of coagulated blood was found between the dura mater and the scull. We removed all that could be scooped out, and the patient partially recovered his sensibility, and the pulse improved. In about an hour afterwards I returned to the case, and introduced a catheter into the bladder, and found it entirely empty.

On the morning of the 25th, the day after the death of the patient, we made an examination of his head. The fracture extended from near the lamdoidal suture through the occipital bone, to the right of the foramen magnum, and terminating in the posterior foramen lacerum. A large mass of coagulated blood was found occupying all the space included in the fracture. The middle meningeal artery had been lacerated ; but why the evidences of this were so obscure, I am ata loss to know. Just before the spasms came on, the patient seized a tumbler of cham pagne brandy, and swallowed the whole of the liquor before the glass could be wrested from him. Had this any connection with the meningeal hemorrhage ? Is it possible that the integrity of the vessel was injured by the fall, and the laceration made complete by the stimulus of the brandy ? These are questions for which I am at a loss for an answer, and they are submitted to the profession for its judgment.

